# Variations in catheter-related bloodstream infections rates based on local practices

**DOI:** 10.1186/2047-2994-2-10

**Published:** 2013-04-03

**Authors:** Soraya Cherifi, Georges Mascart, Anne Dediste, Marie Hallin, Michèle Gerard, Marie-Laurence Lambert, Baudouin Byl

**Affiliations:** 1 Infection Control Unit, Brugmann University Hospital, Place A. Van Gehuchten, 4, Brussels, 1020, Belgium; 2 Microbiology Department, Brugmann Hospital, Brussels, Belgium; 3 Microbiology Department, Saint Pierre Hospital, Brussels, Belgium; 4 Microbiology Department, Erasme University Hospital, Brussels, Belgium; 5 Infection Control Unit, Saint Pierre Hospital, Brussels, Belgium; 6 Healthcare-associated infections unit, Public Health and Surveillance department, Scientific Institute of Public Health, Brussels, Belgium; 7 Infection Control Unit, Erasme University Hospital, Brussels, Belgium; 8 School of Public Health, Université Libre de Bruxelles, Brussels, Belgium; 9 Present address: Centre de Diagnostic Moléculaire, Iris-Lab, Brussels, Belgium

**Keywords:** Catheter-related bloodstream infection, Surveillance, Intensive care unit

## Abstract

**Background:**

Catheter-related bloodstream infection (CRBSI) surveillance serves as a quality improvement measure that is often used to assess performance. We reviewed the total number of microbiological samples collected in three Belgian intensive care units (ICU) in 2009–2010, and we described variations in CRBSI rates based on two factors: microbiological documentation rate and CRBSI definition which includes clinical criterion for coagulase-negative *Staphylococcus* (CNS) episode.

**Findings:**

CRBSI rates were 2.95, 1.13 and 1.26 per 1,000 estimated catheter-days in ICUs A, B and C, respectively. ICU B cultured fewer microbiological samples and reported the lowest CRBSI rate. ICU C had the highest documentation rate but was assisted by support available from the laboratory for processing single CNS positive blood cultures. With the exclusion of clinical criterion, CRBSI rates would be reduced by 19%, 45% and 0% in ICUs A, B and C, respectively.

**Conclusion:**

CRBSI rates may be biased by differences of blood culture sampling and CRBSI definition. These observations suggest that comparisons of CRBSI rates in different ICUs remain difficult to interpret without knowledge of local practices.

## Introduction

Nosocomial bloodstream infections (BSIs) are an important problem worldwide. BSIs comprised 14% of all nosocomial infections in Belgium in 2007 [1]. Catheter-related bloodstream infection (CRBSI) is a leading cause of BSI, particularly in intensive care units (ICUs), where it is associated with significant patient morbidity and mortality and increased health care costs [2]. In 2010, the Belgian National Program for Surveillance of Hospital Infections (NSHI) reported a mean incidence of CRBSI of 1.4 (95% CI: 1.3-1.5) per 10,000 patient-days [3]. The comparison of CRBSI rates between hospitals as a quality indicator and for identification and implementation of prevention strategies remains complex and controversial [4,5]. We hypothesized that even using the same definition, comparing rates of CRBSI between different ICUs may be inappropriate. Other variables, such as the total number of blood cultures obtained and the clinical and laboratory protocols used for the management of blood cultures positive for skin contaminants could have an influence on the rates of CRBSI. This study analyses retrospective data on CRBSI rates from three different ICUs and discusses the limitations of CRBSI surveillance.

## Methods

This retrospective study was conducted in three hospitals in Brussels, Belgium. Hospitals A and B are tertiary teaching hospitals that are affiliated with the same university, i.e., the Université Libre de Bruxelles. Hospitals A and B have 854 and 509 beds, respectively. Hospital C is an academic hospital, and it has 864 beds. Each hospital has an ICU with both medical and surgical critical care beds: 30 beds in ICU-A, 20 beds in ICU-B, and 31 beds in ICU-C. The study population consisted of all patients admitted to the ICUs who developed a CRBSI between January 2009 and December 2010. Cases were retrospectively identified through a search in the microbiological database of each hospital. Only medical records of patients with CRBSI with a single positive percutaneously-drawn blood culture with a skin contaminant were examined further for antimicrobial treatment. We did not collect information regarding severity of illness. In 2011, a six-month prospective surveillance study was conducted in ICUs A and B to measure the CVC utilization ratio (defined as the ratio between catheter-days and patient-days), resulting in a ratio of 0.75. The mean CVC utilization ratio in ICU-C was estimated to be 0.89 (min 0.70, max 1), with 31 point prevalence surveys performed by a local infection control team in 2012.

According to the national surveillance protocol for BSI in Belgium [3], we defined CRBSI as a BSI with: 1) at least one positive blood culture for a recognized pathogen and a positive quantitative CVC tip culture for the same organism or 2a) at least 2 positive blood cultures for a skin contaminant (e.g., coagulase-negative *Staphylococcus* (CNS)) and a positive CVC tip culture or 2b) one single peripheral positive blood culture positive for CNS and a positive CVC tip culture with appropriately instituted antimicrobial therapy. Catheter tips were processed using Maki’s semi-quantitative culture method [6].

The following microbiological data were recorded: number of cultured catheter tips, number of colonized catheter tips, number of blood cultures, number of positive blood cultures, and number of catheter tips removed with concomitant blood cultures. Each hospital had infection control programs and participated in the National Hand Hygiene Campaign in the winter of 2010–2011 [7]. Local procedures for CVC insertion followed standard guidelines, including the use of full sterile barrier precautions during insertion, a 0.5% chlorhexidine alcoholic antiseptic for skin preparation, and hand hygiene. No catheters coated with antimicrobial agents or impregnated with antiseptic were used.

In the laboratories of hospitals A and B, complete strain identification and susceptibility tests were performed on all blood cultures (recognized pathogens and CNS) and catheter tip cultures. In hospital C, species identification of CVC tip cultures was only performed in cases in which one peripheral blood culture (out of two or three) was positive for CNS. If the species were concordant and if the attending physician requested it, an antimicrobial susceptibility profile was then performed, which could take up to 48 hours. Protocols for drawing blood cultures were the same in each of the 3 ICUs: “collection of 2 blood cultures per episode whenever possible using venepuncture and not through an intravascular device”.

Chi-squared tests were used to compare categorical variables. Statistical significance was designated as a 2-sided p*-*value of < 0.05.

## Results

During the 2-year period, using criteria 1, 2a and 2b, 71 episodes of CRBSI occurred during 47,991 patient-days and 38,248 estimated catheter-days: 42 in ICU-A, 11 in ICU-B, and 18 in ICU-C. The numbers of catheters and blood samples cultured per ICU are reported in detail in Table 1.

**Table 1:** Number of catheters and blood samples cultured per intensive care unit

**Intensive care unit (ICU)**	**ICU-A**	**ICU-B**	**ICU-C**	**Total**
**Ratio of positive catheter tips (%)**	123/543 (23%)	48/398 (12%)	89/1,321 (7%)	260/2,262 (11%)
**Catheters tips cultured per 1,000 patient-days**	**29**	**31**	**82**	**47**
**Ratio of positive blood cultures (%)**	1,041/5,578 (19%)	746/4,147 (18%)	845/6,534 (13%)	2,632/16,259 (16%)
**Blood culture rate per 1,000 patient-days**	**291**	**321**	**406**	**339**
**Number of catheter tips cultured with concomitant blood cultures**	489	254	1,094	1,837
**Catheter tips cultured with concomitant blood cultures per 1,000 patient-days**	**26**	**20**	**68**	**38**

The isolated microorganisms are described in Table 2. The microorganism most frequently isolated was S*taphylococcus epidermidis* (n = 17). The ratio of CRBSI-documented infections (number of blood cultures with concomitant catheter tip cultures) was also statistically different between the 3 ICUs (*P <* 0.001). This result remained significant if the number of blood cultures was used instead of patient-days.

**Table 2:** Distribution of microorganisms isolated from CRBSIs

**Microorganisms**	**ICU-A**	**ICU-B**	**ICU-C**	**Total**
**Gram-positive cocci**	**23**	**10**	**8**	**41**
Coagulase-negative *Staphylocci*	13	9	2	24
*Staphylococcus aureus*	9	0	4	13
*Enteroccocus sp.*	1	1	2	4
**Gram-negative bacilli**	**15**	**1**	**9**	**25**
*Enterobacteriaceae*	12	1	5	17
Other Gram-negative Bacilli	3	0	4	7
**Yeasts**	**4**	**0**	**1**	**5**
**Total**	**42**	**11**	**18**	**71**

The CRBSI rates were 2.95, 1.13 and 1.26 per 1,000 estimated catheter-days in ICUs A, B and C, respectively. There were a total of 8 single CNS positive blood cultures in ICU-A (all treated with antibiotics), 7 in ICU-B (5 treated), and 12 in ICU-C (none treated). CRBSI rates based on the exclusion of criterion 2b were 2.39, 0.62 and 1.26 per 1,000 estimated catheter-days in ICUs A, B and C, respectively. The ratios of CRBSIs was statistically different between the 3 ICUs (*P* = 0.003), even when criterion 2b was removed (*P* = 0.003).

With the exclusion of criterion 2b (if all CNS BSIs were processed according to laboratory routine C and/or managed with a strict reduction in initiation of antibiotic treatment), the ranking of CRBSIs would not be modified; however, a reduction of CRBSI rates of 19% and 45% in ICU-A and -B, respectively, would be seen.

## Discussion

Benchmarking of surveillance data for health-care-associated infections, especially for central line-associated bloodstream infections (CLABSI), has been used for more than two decades to identify prevention strategies and to improve patient safety.

The criteria used to define intravascular catheter-related infection are difficult to apply; perhaps more simplified methods of surveillance that rely entirely on objective criteria would be a better option [8]. CLABSI refers to a primary BSI in a patient who had a central venous line within the 48-hour period prior to development of the BSI that was not related to an infection at another site [9]. A secondary BSI from an undocumented source could be erroneously labelled a CLABSI, overestimating the true incidence [10]. In Belgium, the national surveillance protocol for healthcare-associated BSIs makes a distinction between “proven” or “probable” CLABSI [3]. These confusing definitions increase the ambiguity in classification and justify the use of the CRBSI definition instead of the CLABSI definition in this retrospective study. In reality, measurement of catheter-days is relatively time consuming and not routinely performed. In addition, infection control departments have different interpretations of criteria for calculating catheter-days, as demonstrated by Niedner et al. [11]. However, it may be reasonable to assume that estimation of catheter-days will result in a smaller impact on the rates of CRBSI [12-14].

We examined two factors that are known to impact measured CRBSI rates: microbiological documentation rate and definition including clinical criterion influenced by individual local practices.

Inappropriate CVC tip and blood cultures generate a large workload and are a poor use of laboratory resources. In the 3 ICUs, many tips were cultured without concomitant blood cultures. Numerous studies have shown that 15 to 25% of short-term CVC cultures are colonized, most commonly by CNS, though most patients show no evidence of infection [15,16]. Differences in microbiological documentation make comparisons between ICUs difficult to interpret. The total number of blood cultures sampled in ICU-C was higher than in ICUs A and B, higher than the national average of 42 per 1,000 patient-days (including non-ICU patients) [3] and higher than the median of 73 per 1,000 patient days reported in European countries [17]. The considerable number of microbiological analyses done in ICU-C might be justified, as the patients in ICU-C, who included transplant patients, haematological patients at high risk of complications, and patients who were transferred from others hospitals, are more likely to have severe health problems. In contrast, the total number of blood cultures performed in ICU-B with concomitant catheter-tip cultures was lower than in ICUs A and C. ICU-B also had the lowest CRBSI rate. The number of blood cultures performed could have a significant influence on CRBSI rates, as demonstrated by Gastmeier et al. [18]. Niedner et al. found a significant correlation between a surveillance aggressiveness score and higher CRBSI rates [11]. We did not collect clinical information that could explain the high CRBSI rate in ICU-A. However, both ICUs A and B managed patients that were usually less complicated than those treated in the academic hospital.

Today, CNS is recognized as the most common cause of CRBSI, consistent with the results from our study. Local laboratory practices (incomplete identification of single CNS blood culture) could influence how the physician decides whether a single peripheral CNS-positive blood culture is a contaminant. It could explain how zero CRBSI episodes fulfilled criterion 2b in ICU-C. The clinical importance of a single CNS positive blood culture also depends on physician training and experience. Moreover, an uncomplicated CRBSI caused by CNS may not necessarily require a treatment [19]. The use of clinical criterion (criterion 2b) introduces a bias that makes comparisons very difficult*.* In ICUs A and B, treatment of patients with a possibly contaminated blood culture combined with catheter colonization could have accounted for a certain number of CNS CRBSI. However, in our national protocol of BSI surveillance, CNS BSI represented a minority (14%) of the total reported BSIs, and among this group, CNS BSI with at least 2 positive blood cultures constituted the majority (79%) (3). The CDC CLABSI definition changed beginning in 2008, correctly removing criterion 2b [20].

Our study has several limitations. First, the results are limited by the study’s retrospective nature and by the small sample size. Second, we did not collect any clinical data except for the administration of antibiotic treatment. Finally, the number of catheter-days was estimated with two different methods and at different periods. Therefore, comparison of CRBSI rates between ICUs might have been distorted.

## Conclusion

Our study describes the variation in measured CRBSI rates in 3 neighbouring hospitals based on laboratory processes and clinical practices. Interpretation of “raw” data on hospital performance is dependent on many variables that are beyond the epidemiologist’s control.

Better prospective studies are needed to improve the quality and reproducibility of surveillance definitions and to identify the best practice for a benchmark approach.

## Competing interests

The authors declare that they have no competing interests.

## Authors’ contributions

SC and BB were responsible for the study concept and design. SC, GM, AD, and MH performed data collection. SC, BB, MG, and MH were responsible for data analysis. All authors were involved in drafting the manuscript or revising critically for important intellectual content. All authors have read and approved the manuscript.
